# Epicardial Atrial Fat at Cardiac Magnetic Resonance Imaging and AF Recurrence after Transcatheter Ablation

**DOI:** 10.3390/jcdd11050137

**Published:** 2024-04-28

**Authors:** Andrea Ballatore, Marco Gatti, Serena Mella, Davide Tore, Henri Xhakupi, Fabio Giorgino, Andrea Saglietto, Ludovica Carmagnola, Edoardo Roagna, Gaetano Maria De Ferrari, Riccardo Faletti, Matteo Anselmino

**Affiliations:** 1 Division of Cardiology, Cardiovascular and Thoracic Department, “Città della Salute e della Scienza” Hospital, 10126 Turin, Italyludovica.carmagnola@unito.it (L.C.); edoardo.roagna@unito.it (E.R.);; 2 Department of Medical Sciences, University of Turin, 10126 Turin, Italy; mella.serena1998@gmail.com; 3 Radiology Unit, Department of Diagnostic Imaging and Interventional Radiology, “Città della Salute e della Scienza” Hospital, 10126 Turin, Italy; m.gatti@unito.it (M.G.); riccardo.faletti@unito.it (R.F.); 4 Department of Surgical Sciences, University of Turin, 10126 Turin, Italy; 5 Dipartimento di Medicina Interna, Università Degli Studi di Genova, 16126 Genoa, Italy

**Keywords:** atrial fibrillation, catheter ablation, epicardial adipose tissue, magnetic resonance, cryoballoon ablation

## Abstract

The relationship between epicardial adipose tissue (EAT) and atrial fibrillation (AF) has gained interest in recent years. The previous literature on the topic presents great heterogeneity, focusing especially on computed tomography imaging. The aim of the present study is to determine whether an increased volume of left atrial (LA) EAT evaluated at routine pre-procedural cardiac magnetic resonance imaging (MRI) relates to AF recurrences after catheter ablation. A total of 50 patients undergoing AF cryoballoon ablation and pre-procedural cardiac MRI allowing quantification of LA EAT were enrolled. In one patient, the segmentation of LA EAT could not be achieved. After a median follow-up of 16.0 months, AF recurrences occurred in 17 patients (34%). The absolute volume of EAT was not different in patients with and without AF recurrences (10.35 mL vs. 10.29 mL; *p*-value = 0.963), whereas the volume of EAT indexed on the LA volume (EATi) was lower, albeit non-statistically significant, in patients free from arrhythmias (12.77% vs. 14.06%; *p*-value = 0.467). The receiver operating characteristic curve testing the ability of LA EATi to predict AF recurrence after catheter ablation showed sub-optimal performance (AUC: 0.588). The finest identified cut-off of LA EATi was 10.65%, achieving a sensitivity of 0.5, a specificity of 0.82, a positive predictive value of 0.59 and a negative predictive value of 0.76. Patients with values of LA EATi lower than 10.65% showed greater survival, free from arrhythmias, than patients with values above this cut-off (84% vs. 48%; *p*-value = 0.04). In conclusion, EAT volume indexed on the LA volume evaluated at cardiac MRI emerges as a possible independent predictor of arrhythmia recurrence after AF cryoballoon ablation. Nevertheless, prospective studies are needed to confirm this finding and eventually sustain routine EAT evaluation in the management of patients undergoing AF catheter ablation.

## 1. Introduction

Consideration of the role of epicardial adipose tissue (EAT) in the development of atrial fibrillation (AF) has increased in recent years. EAT refers to that portion of cardiac visceral adipose tissue in direct contact with the visceral layer of the pericardium above the myocardium. It has peculiar characteristics that differentiate it from the other types of visceral adipose tissue; EAT shares properties with both white and brown adipose tissue, so the term “beige” adipose tissue has been proposed to appropriately define it [1]. EAT plays several roles in the regulation of cardiac function and has a profound and complex interaction with the heart: it represents a storage of energy resources for the cardiac cells (in the older age), and it has thermogenesis properties (in the youngest) [2]; through endocrine and paracrine interactions, it mediates inflammatory response and modulates the immune system [3,4]; finally, ganglionated plexi, key structures of the autonomic nervous system of the heart, are located in the EAT.

An excess or dysregulation of EAT has been shown to be involved in the pathogenesis of heart failure with a preserved ejection fraction [5,6] and coronary artery disease through an imbalance in the inflammatory state that promotes atherogenesis and the development of unstable plaques [7,8]. The exact pathophysiology of the interplay between EAT and AF is not yet entirely clarified: dysfunctional or excessive EAT through the secretion of adipokines and cytokines favors an inflammatory milieu and the production of reactive oxygen species, which, in turn, can promote fibrogenesis; on the other hand, it has been postulated that its proarrhythmic effects are mediated by modification in the expression and function of cardiac ion channels, resulting in arrhythmogenic electrical remodeling [9,10,11]. Indeed, classic pro-inflammatory cytokines—such as TNF-alpha, IL-6 and IL-1 beta—as well as components of the inflammasome and of the immune response, are overly expressed in the dysregulated EAT [12]. Moreover, the free fatty acid infiltration of atrial myocytes and increased parasympathetic activity lead to increased dispersion of action potential duration and, subsequently, facilitate the occurrence of micro-reentry [13].

In summary, excessive EAT can be a mediator in the development of atrial cardiomyopathy, a complex disorder inducing an imbalance in normal atrial physiology associated with endothelial dysfunction, atrial arrhythmias and increased thromboembolic risk [14]. Clear and unique imaging criteria for the diagnosis of atrial cardiomyopathy are not well defined [15]: transthoracic echocardiography is the most common imaging modality to assess LA dimension, but its use for the evaluation of LA function (e.g., LA strain) is limited by image quality and acoustic window; nevertheless, LA strain has been associated with AF recurrence after ablation [16] and negative cardiovascular outcomes, especially in women [17,18]. On the contrary, cardiac MRI allows a reproducible quantification of LA dimension, tissue characterization and fibrosis quantification, including functional assessment analogous to echocardiographic speckle tracking [19].

Catheter ablation is an effective and safe option for the treatment of AF and has earned strong recommendations in guidelines for rhythm control [20], as well as a first-line option [21]. Nevertheless, the correct selection of the patients is fundamental in order to avoid referring to an invasive procedure patient who has a high risk of recurrences.

The aim of the present study is to determine whether an increased volume of atrial EAT evaluated at routine pre-procedural imaging relates to the recurrence of AF after catheter ablation.

## 2. Materials and Methods

All patients undergoing AF cryoballoon transcatheter ablation from January 2019 to December 2021 with a preprocedural cardiac magnetic resonance (MRI) with sequences allowing for epicardial adipose tissue quantification were retrospectively included. A total of 50 patients were finally included in the analysis. At our center, in order to limit X-ray exposure deriving from other imaging techniques, all patients who did not present cardiac MRI contraindications underwent preprocedural LA-focused MRI before ablation instead of contrast-enhanced cardiac computed tomography (CT) scan. Key exclusion criteria were age < 18 years, any contraindication to MRI (claustrophobia, severe obesity, allergy to contrast medium and/or eGFR < 30 mL/min/1.73 m^2^) and unwillingness to sign informed consent. All enrolled patients signed a written informed consent. Arrhythmia recurrences were defined as ECG-documented recurrences at 12-lead ECG and 24 h Holter recordings or as clinically recognized symptomatic episodes. All patients were routinely followed up with 24 h ECG Holter recording at 3 and 12 months, outpatient visits and telephonic interviews. The study was approved by the local ethical committee and conducted according to the declaration of Helsinki.

Before the ablation procedure, all patients underwent transesophageal echocardiography (TEE) to rule out left atrial appendage (LAA) thrombosis and cardiac MRI to assess LA anatomy. A 1.5 T scanner (Achieva, version 2.6, Philips Medical Systems, Eindhoven, The Netherlands) using a 32-channel body phased-array coil was used. Paramagnetic contrast medium (Prohance, gadoteridol, Bracco Imaging, Milan, Italy) was administered at a dose of 0.1 mmol/kg. The sequences acquired during the exam were as follows: Angio-MRI (non-ECG-gated, 4D time-resolved with keyhole 4D-TRAK); LGE-MRI (free breathing navigator and ECG-gated inversion recovery gradient-echo sequence). The ablation protocol adopted has been previously detailed elsewhere [22]. Briefly, through venous femoral accesses, a diagnostic decapolar electrophysiological catheter was placed in the coronary sinus (CS); subsequently, access to the LA was achieved through a patent foramen ovale or by single transseptal puncture and a 28 mm cryoballoon ablation catheter (Arctic Front Advance, Medtronic, Minneapolis, MN, USA) and an inner lumen mapping catheter (Achieve, Medtronic) via a steerable 15 Fr sheath (FlexCath Advance, Medtronic) were advanced in each pulmonary vein (PV) ostium. An activated clotting time >350 s was maintained during the procedure. According to our standard approach, cryoenergy was delivered at each PV ostium for 180 s if PV isolation was achieved in less than 60 s for 240 s in the other cases. Cryoenergy application was immediately stopped if the temperature reached −55 °C or in case of loss of diaphragmatic stimulation during right PVs ablation. Acute PVI was confirmed during index ablation by demonstrating the absence of PV potentials after the cryoenergy applications on the Achieve (Medtronic) inner lumen circular mapping catheter.

Volume of left atrial epicardial adipose tissue on the acquired images was manually quantified, not independently, by two trained radiologists (M.G and D.T.), who were blinded to the recurrence status of the patients. Manual segmentations were performed on LGE-MRI images using open-source software 3D Slicer (https://www.slicer.org/) (accessed at 10 March 2024).

The primary outcome of the study was to correlate left atrial adipose tissue, evaluated at cardiac MRI, with recurrence after AF cryoballoon ablation. Both absolute LA epicardial adipose tissue volume and LA epicardial adipose volume tissue indexed on the LA volume (henceforth referred to as LA EATi) were considered for the analyses. The latter value was obtained by dividing the LA EAT volume by the absolute LA volume.

### Statistical Analysis

Categorical variables are reported as percentages. Continuous variables are reported as mean ± standard deviation (SD) or median and interquartile range (IQR). Student’s *t*-test and Fisher exact test were used to compare continuous and categorical variables between groups, respectively. A receiver operating characteristic (ROC) curve was used to analyze the relationship between AF recurrences and the absolute LA EAT volume and the LA EAT volume indexed on the LA volume (LA EATi). The strength of the predictive ability of the variables in exam was evaluated with the area under the curve (AUC): a value greater than 0.7 was considered indicative of an acceptable association. Youden index was used to identify the best cut-offs at ROC analyses. Survival analyses were performed with Kaplan–Meier curves and log-rank test comparing two groups stratified on the cut-off identified at the ROC analysis. Univariate and multivariate Cox regression analyses were used to identify predictors of AF after ablation; clinical and radiological predictors (gender, age, hypertension, diabetes, previous stroke, thyroid disease, valvular disease, diabetes, duration of AF history, body surface area, beta-blockers use, flecainide use, LA EATi ≥ 10.65% and BMI) were included in the univariate model. A multivariate analysis including diabetes, age, body surface area, LA EATi ≥ 10.65% and BMI was also performed. These parameters were chosen clinically due to their potential effects in altering the relationship between LA EAT and AF recurrence after catheter ablation and in reflecting the metabolic status of the patients. A two-tailed *p* value < 0.05 was considered statistically significant. All analyses were performed using R software version 4.2.2 (R Foundation for Statistical Computing, Vienna, Austria).

## 3. Results

Segmentation and quantification of LA EAT volume at cardiac MRI could not be achieved in one case out of the 50 enrolled patients. Baseline characteristics of the study population are reported in Table 1. The mean age was 59.6 years (±11.0 years), 38% of patients were females and the majority presented paroxysmal AF (86%); the mean number of previous AADs (effective, not-effective or not-tolerated) or rate control agents was 1.7 per patient. At baseline echocardiography mean ejection fraction was 61%. Mean BMI was 26.0 Kg/m^2^ (±3.1 Kg/m^2^), with only 10% of patients affected by obesity. At MRI, mean indexed LA volume was 43.3 mL/m^2^ (±12.3 mL/m^2^). All patients underwent, at first, PVI ablation procedures; four patients had already undergone cavo-tricuspid isthmus ablation for typical atrial flutter. After a median follow-up of 16.0 months (IQR: 12.0–21.8), AF relapsed in 17 patients (34%). For two patients, both highly symptomatic, AF recurrence was defined on the basis of symptoms (similar to those prior to AF ablation, and in one of the two cases, symptoms receded after oral flecainide assumption). In all other cases, AF recurrence was documented electrocardiographically, according to the AF definition of current guidelines. No statistically significant differences in baseline characteristics were found between patients with and without recurrences, with the exception of greater use of beta-blockers at baseline (79% vs. 47%, *p*-value = 0.019) in patients free from arrhythmic recurrences. No difference in the use of AADs during follow-up was observed.

Overall, acute PVI was obtained in 97.8% of cases. No difference in acute PVI was observed between patients with and without AF recurrence at follow-up (93.7% vs. 98.3%, *p* = 0.896).

**Figure 1:**
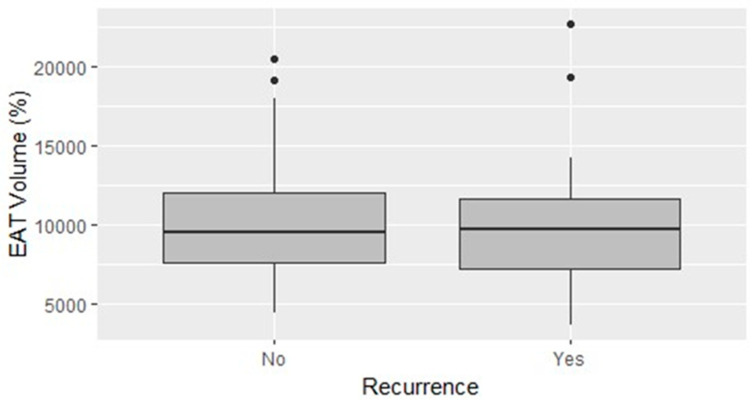
Direct comparison of the absolute LA EAT volume according to recurrence status.

**Figure 2:**
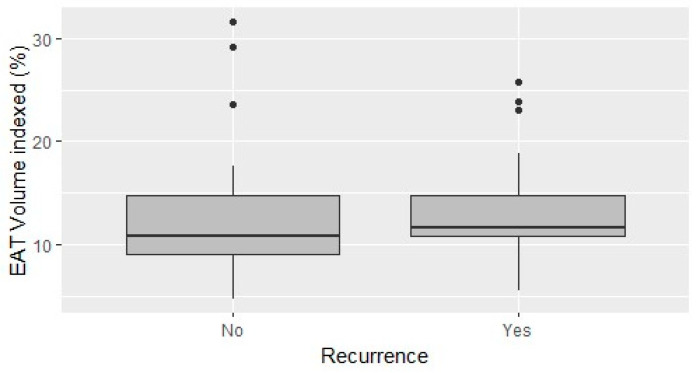
Direct comparison of the LA EATi volume according to recurrence status.

**Figure 3:**
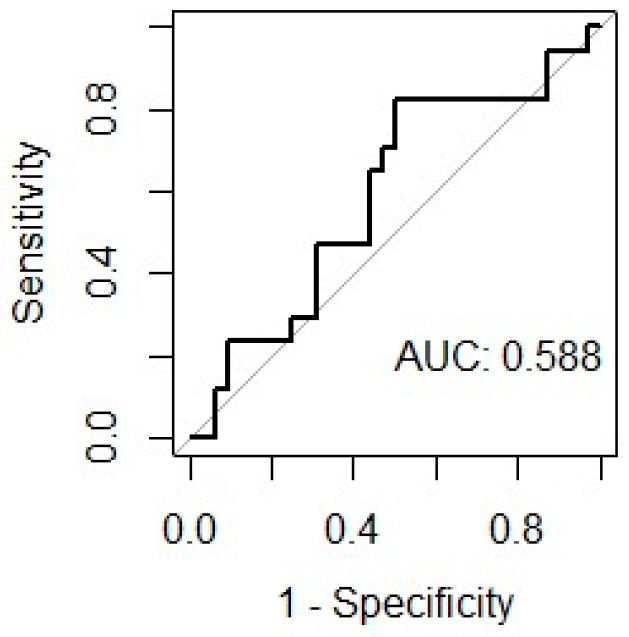
ROC curve for the LA EATi.

**Figure 4:**
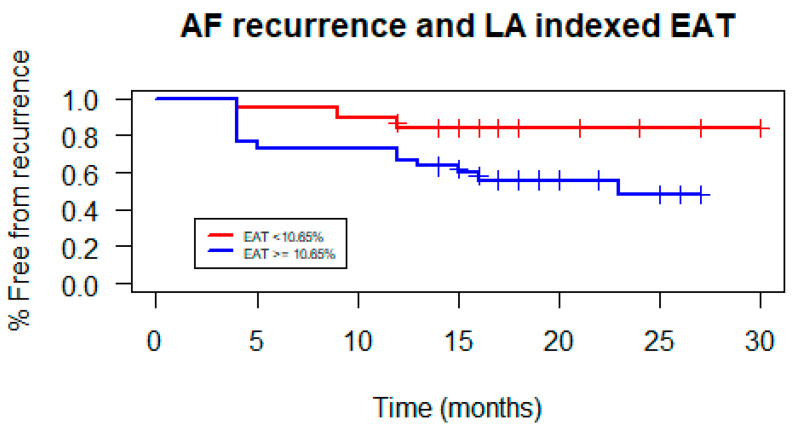
Kaplan–Meier arrhythmia-free survival curves stratified by the LA EATi above or below 10.65%.

The mean EAT volume was 10.3 mL, and the EAT volume indexed on the LA volume (LA EATi) was 13.2%. The absolute volume of EAT did not diverge in patients with and without AF recurrences (10.35 mL vs. 10.29 mL; *p*-value = 0.963; Figure 1), whereas the LA EATi was lower, albeit non-statistically significant, in patients not suffering relapses during follow-up (12.77% vs. 14.06%; *p*-value = 0.467) (Figure 2). The ROC curve testing the ability of LA EATi to predict AF recurrence after cryoballoon ablation (Figure 3) showed a sub-optimal performance (AUC: 0.588). The finest identified cut-off, able to predict patients with AF recurrence, was 10.65% with a sensitivity of 0.5, a specificity of 0.82, a positive predictive value of 0.59 and a negative predictive value of 0.76. The absolute proportion of patients with AF recurrence was lower, albeit non-formally statistically significant, in patients with an LA EATi inferior to 10.65% compared to those with a value above the cut-off (15.8% vs 46.7%, respectively, p= 0.057). Patients with an LA EATi value lower than the identified cut-off (10.65%) showed greater survival free from arrhythmias than patients with a value above the cut-off (recurrence rate 84% vs. 48%; p-value=0.04; Figure 4). Figures S1 and S2 (see Supplementary Material) illustrate the ROC curve and subsequent Kaplan–Meier analysis stratified by absolute LA EAT volume.

Table 2 shows univariate and multivariate Cox regression analyses for AF recurrences as previously described. At multivariate analysis, LA EATi above the cut-off of 10.65% was independently associated with an increased risk of AF recurrences (HR 4.5; 95%CI: 1.2–17.3; adjusted for BSA, diabetes, age, BMI and beta-blockers use). Diabetes also emerged as an independent risk factor of cryoballoon catheter ablation failure (HR 6.2; 95%CI:1.3–29.4), whereas beta-blockers showed a protective effect (HR 0.31; 95%CI: 0.10–0.98).

## 4. Discussion

The main findings of the present study are as follows:

- Evaluation of left atrial adipose tissue by means of routine preprocedural cardiac MRI imaging is feasible;
- Levels of left atrial adipose tissue volume indexed on LA volume are associated with a higher risk of arrhythmic recurrence after AF cryoballoon catheter ablation.

The identification of the optimal candidates for catheter ablation of AF is of paramount importance. The assessment of the risk of AF recurrence after the procedure is a fundamental step in the management of patients with AF. More importantly, in the case of modifiable risk factors, such as obesity, patients can be encouraged to improve lifestyle habits, delaying ablation when and whether these risk factors have been controlled. Recently, web-based risk scores have been made freely available to physicians and patients to predict the probability of AF recurrence after catheter ablation (AFA-RECUR) [23]. Interestingly, BMI has a deep impact on the risk of arrhythmic recurrence, and reduction in body weight is associated with a similar decrease in arrhythmic risk. In our cohort, nevertheless, BMI was not different, nor did it predict arrhythmic recurrence after catheter ablation, probably because of the low prevalence of obesity in the enrolled population and the fairly homogenous values of BMI.

Bearing in mind the recent extension of gliflozin use for the treatment of heart failure, greater attention to the metabolic aspects should also be posed for AF patients: indeed, SGLT2 inhibitors have shown to reduce the risk of new-onset AF along with a reduction in body weight [24,25]. These effects are, at least partially, mediated by a reduction of epicardial adipose tissue. Another new oral antidiabetic drug, semaglutide, recently showed weight-lowering properties, but its effect on the prevention of atrial arrhythmias is still unclear [26]. On these bases, the evaluation of left atrial adipose tissue may play an important role in the management of patients with AF, both in adding a parameter to assess in search of the likelihood of AF recurrence and in identifying the correct timing of the procedure once other risk factors are controlled, in order to avoid futile procedures.

The previous literature on the topic presents great heterogeneity in both the methods of EAT quantification and the parameters analyzed. Overall, several studies focused on cardiac EAT, with different approaches and definitions: the heterogeneity in methodology resulted in somewhat contradictory results [27,28]. A recent report by Hammache et al. evaluated the association between EAT measured by CT scan and arrhythmic recurrence after ablation of paroxysmal AF; no association between total EAT volume or EAT density was found with AF recurrence [28]. Indeed, in a previous experience, LA EAT was only associated with AF recurrence, whereas total EAT did not relate to ablation failure [29]. Notwithstanding, a growing body of evidence, clinical and pathophysiological, supports the hypothesis that EAT is implied in AF development. Our study adds further insights into this relationship, specifically focusing on LA EAT and its role in AF recurrence after cryoballoon ablation.

The preferred imaging modality adopted in previous studies was cardiac CT, whereas cardiac MRI has been seldom used; thickness of EAT, as well as total and LA volume of EAT, have been proposed. Indeed, the recent consensus document on AF ablation supports the use of cardiac CT as preprocedural imaging for the evaluation of EAT [30]; however, it stresses, on the other hand, the role of cardiac MRI in fibrosis quantification. A previous meta-analysis by Shamloo [27] summarized current evidence by grouping studies on the basis of the variable analyzed: the Authors found a significant difference in LA EAT volume, analyzed at CT, in patients with and without AF recurrences after catheter ablation; however, the difference was in crude values, not corrected for other risk factors that may have potentially driven the association. A more recent systematic review [31] meta-analyzed the relative risk and hazard ratio of several EAT parameters and AF recurrence after catheter ablation, suggesting that relative EAT thickness but not EAT volume measures are related to an increased risk of recurrence. More specifically, this relation was more evident in younger patients (less than 60 years old), the Asian AF population, and in the case of longer follow-ups. These discrepancies may be explained by the fact that atrial cardiomyopathy and, therefore, the level of fatty infiltration and the burden of epicardial adipose tissue are less likely in young, healthier patients. In the latter cohorts, in fact, the variance in LA EAT volume among distinct patients and its possible role on AF recurrence may be attenuated and more difficult to statistically detect. On the other hand, in such a scenario, the role of epicardial adipose tissue, in the case of evidently high-fat deposit burden, may be the most relevant for the development of AF recurrence after ablation. Older and sicker patients, who commonly present the highest levels of LA EAT, may, in fact, present several competitive risk factors for AF recurrence besides LA EAT: in these cases, the isolated role of LA EAT in conferring a higher risk of AF recurrence after catheter ablation may also be difficult to detect.

Only a few works have focused on the evaluation of LA EAT with cardiac MRI [24,32]. Indeed, cardiac MRI holds the potential to accurately assess the characteristics of atrial cardiomyopathy, of which EAT is a part. Tissue characterization and fibrosis quantification are important indicators of the severity of atrial remodeling. Nevertheless, there is still some discrepancy in the amount of LA fibrosis quantification among different methodologies, and the benefit of fibrosis-guided ablation is debated [22,33,34]. LA atrial function can be accurately measured with an analysis of the volumes and MRI-derived myocardial feature tracking quantifying the deformation process of the atrium as echocardiographic speckle tracking. Finally, novel parameters are being evaluated as markers of atrial disease and atrial cardiomyopathy: the sphericity index, derived from the asymmetric process of LA dilatation, has been proposed as a predictor of increased LA pressure, but it recently failed to demonstrate an association with the type of AF [35]. The accurate analysis of flow dynamics inside the LA by means of cardiac MRI and 4D framework are novel methods for the assessment of LA function [36]; the assessment of PV morphology and flow is a promising area of research but is still in need of validation [15].

**Figure 5:**
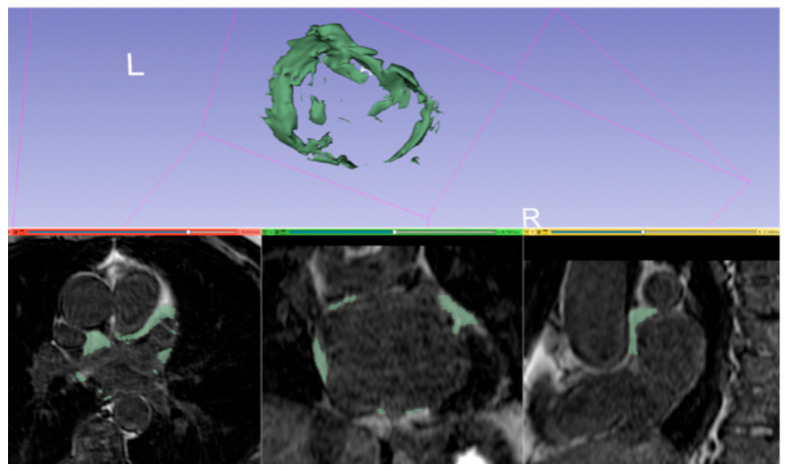
Example of segmentation and 3D reconstruction of LA EAT at cardiac MRI. L: left; R: right.

The present study focused exclusively on the analysis of epicardial adipose tissue at the left atrium level (Figure 5): due to the reduced left atrial thickness, the distinction between epicardial and pericardial adipose tissue is challenging and could have entailed a less accurate quantification; on the other hand, left atrial epicardial adipose tissue embraces ganglionated plexi, which play an important role in AF arrhythmogenesis. Indeed, we found that an amount of LA EAT volume equal or superior to 10.65% of the total volume of the LA was independently associated with the risk of AF recurrence after cryoballoon ablation. These findings are consistent with those recently published by Chahine et al. with a different cut-off [24]; however, the present study differs from the latter in several aspects: first, atrial EAT was quantified by 3D-LGE and not Dixon sequences, the former being widely known and easily implementable in a routine pre-procedural exam. Second, the study by Chahine et al. included patients who mostly underwent radiofrequency ablation. On the contrary, we included patients who exclusively underwent cryoballoon ablation in order to assess the effect of EAT in this particular scenario. Moreover, in the latter study, patients had more often non-paroxysmal AF, higher BMI, higher LA volume indexed for BSA at baseline and were older, indicating a different population with probably a more advanced disease.

Both studies examined LA EAT volume, but Chahine et al. normalized for the body surface area instead of LA volume. Normalizing for LA volume allows us to eliminate the influence that a larger atrium may have on EAT independently from BSA.

The quantification of atrial adipose tissue by means of cardiac MRI is surely feasible if the preprocedural imaging is focused on LA, without the need for the acquisition of the whole cardiac volume, significantly shortening examination time. From a practical point of view, this is very important as it allows the implementation of a routine evaluation of this parameter without changing the current protocol or adding further time-consuming acquisitions.

### Limitations

This work presents the following limitations. The population in the exam is limited and includes mostly patients with paroxysmal AF, preventing the generalization of the results to the entire AF population. The low numerosity may result in statistical under-power preventing the detection of significant differences. Given the limited sample size, a formal interobserver and/or intraobserver variability measurement would, in any case, not achieve an irrefutable outcome. Nevertheless, several findings of this study are confirmatory of previous reports, such as the recurrence rate of patients with paroxysmal AF after a single catheter ablation, supporting the reliability of the investigated sample. The results of this study need to be considered as confirmatory of previous (similarly populated) studies. The population included in this study comprehends mostly young and otherwise healthy individuals; therefore, it was not possible to explore the possible effects of other comorbidities on AF recurrence. In our cohort, all patients underwent cryoballoon ablation; therefore, it is unknown whether the association will remain valid in the case of another ablation energy.

## 5. Conclusions

LA EAT volume indexed on the LA volume independently related to arrhythmia recurrence after AF cryoballoon catheter ablation. Even if cardiac CT is the most common imaging methodology adopted, as indicated in a recent consensus document, cardiac MRI with LGE sequence appears to be an appropriate methodology for the quantification of LA EAT volume, on top of fibrosis quantification. Prospective studies with larger cohorts are warranted to assess the role of the evaluation of LA EAT volume for the referral of patients to catheter ablation.

## Figures and Tables

**Table 1:** Baseline characteristics of the included patients stratified by arrhythmia recurrence. The reported *p*-value refers to the comparison between recurrence subgroups.

Variable	General Population (n = 50)	Arrhythmic Recurrence (n = 17)	No Recurrence (n = 33)	*p*-Value
Age (years ± SD)	59.64 (±11.0)	59.4 (±12.3)	59.8 (±10.5)	ns
Gender (female)	19 (38%)	8 (47%)	11 (33%)	ns
BMI (kg/m^2^ ± SD)	26.0 (±3.1)	26.0 (±3.9)	26.0 (±2.7)	ns
Obesity	5 (10%)	3 (18%)	2 (6%)	ns
Paroxysmal AF	43 (86%)	17 (100%)	26 (79%)	ns
AF history duration (months ± SD)	66.5 (±81.0)	85.5 (±93.6)	57 (±73.6)	ns
Hypertension	26 (52%)	10 (59%)	16 (48%)	ns
Diabetes	3 (6%)	3 (18%)	0 (0%)	ns
Previous stroke	4 (8%)	2 (12%)	2 (6%)	ns
CAD	1 (2%)	1 (6%)	0 (0%)	ns
Thyroid disorders	6 (12%)	2 (12%)	4 (12%)	ns
*Prior use of AADs*				
Amiodarone	3 (6%)	0 (0%)	3 (9%)	ns
Flecainide	27 (74%)	12 (71%)	25 (76%)	ns
Propafenone	12 (24%)	6 (35%)	6 (18%)	ns
Sotalol	6 (12%)	3 (18%)	3 (9%)	ns
Beta-blockers	34 (68%)	8 (47%)	26 (79%)	0.019
Digoxin	1 (2%)	0 (0%)	1 (3%)	ns
*Oral anticoagulants*				ns
VKA	2 (4%)	0 (0%)	2 (6%)	
DOAC	32 (64%)	12 (71%)	20 (61%)	
LA indexed volume (mL/m^2^ ± SD)	43.3 (±12.3)	39.4 (±10.4)	45.2 (±12.9)	ns
*AAD use during FU*				
Amiodarone	3 (6%)	2 (11%)	1 (3%)	ns
Flecainide	15 (31%)	5 (29%)	10 (31%)	ns
Propafenone	4 (8%)	2 (12%)	2 (6%)	ns
Sotalol	5 (10%)	4 (24%)	1 (3%)	ns
Beta-blockers	25 (51%)	5 (29%)	20 (63%)	ns
Digoxin	1 (2%)	1 (6%)	0 (0%)	ns

AAD: anti-arrhythmic drugs; AF: atrial fibrillation; CAD: coronary artery disease; DOAC: direct oral anticoagulation; FU: follow-up; LA: left atrium; ns: non-significant; SD: standard deviation; VKA: vitamin K antagonist.

**Table 2:** Univariate and multivariate analyses of predictors of AF recurrences reporting HR and 95%CI.

Variable	Univariate	Multivariate
Hypertension	1.4 (0.5–3.7)	
Diabetes	3.9 (1.1–13.9)	6.2 (1.3–29.4)
Previous stroke	1.9 (0.4–8.3)	
Thyroid disease	1.5 (0.5–4.4)	
Valvular disease	3.9 (0.9–17.1)	
Beta-blockers	0.5 (0.2–1.3)	0.3 (0.1–0.98)
Flecainide	1.0 (0.6–1.7)	
Gender	1.6 (0.6–4.3)	
LA EATi ≥ 10.65%	3.3 (1.0–11.7)	4.5 (1.2–17.3)
BSA	0.3 (0.0–5.2)	0.1 (0.0–3.8)
Age	1.0 (1.0–1.0)	1.0 (0.9–1.0)
AF history (months)	1.0 (1.0–1.0)	
BMI (kg/m^2^)	1.0 (0.8–1.2)	1.1 (0.9–1.2)

## Data Availability

The data underlying this article will be shared upon reasonable request to the corresponding author.

## Supplementary Materials

The following supporting information can be downloaded at: https://www.mdpi.com/article/10.3390/jcdd11050137/s1: Figure S1: ROC curve analysis for the absolute LA EAT volume. Finest cut-off identified with the Youden index was 19.2 mL with a sensitivity of 0.12, a specificity of 0.97, positive predictive value of 0.67 and negative predictive value of 0.68; Figure S2: Kaplan–Meier arrhythmia-free survival curves stratified by the absolute LA EAT volume above or below 19.2 mL, showing no statistically significant difference (62% vs. 33%; *p*-value = 0.2).
