# Supplementary material for: Epicardial Atrial Fat at Cardiac Magnetic Resonance Imaging and AF Recurrence after Transcatheter Ablation
Source: J Cardiovasc Dev Dis. 2024 Apr 28;11(5):137. doi: 10.3390/jcdd11050137 (PMC11122251; doi:10.3390/jcdd11050137)

# Epicardial Atrial Fat at Cardiac Magnetic Resonance Imaging and AF Recurrence after Transcatheter Ablation

*Supplementary Material*

**Figure S1**. ROC curve analysis for the absolute LA EAT volume. Finest cut-off identified with the Youden index wass 19.2 ml with a sensitivity of 0.12, a specificity of 0.97, positive predictive value of 0.67 and negative predictive value of 0.68.

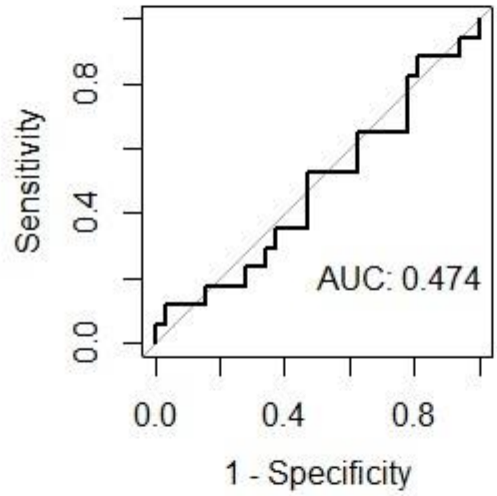

**Figure S2**. Kaplan-Meier arrhythmia-free survival curves stratified by the absolute LA EAT volume above o below 19.2 ml, showing no statistically significant difference (62% vs 33%; p-value=0.2).

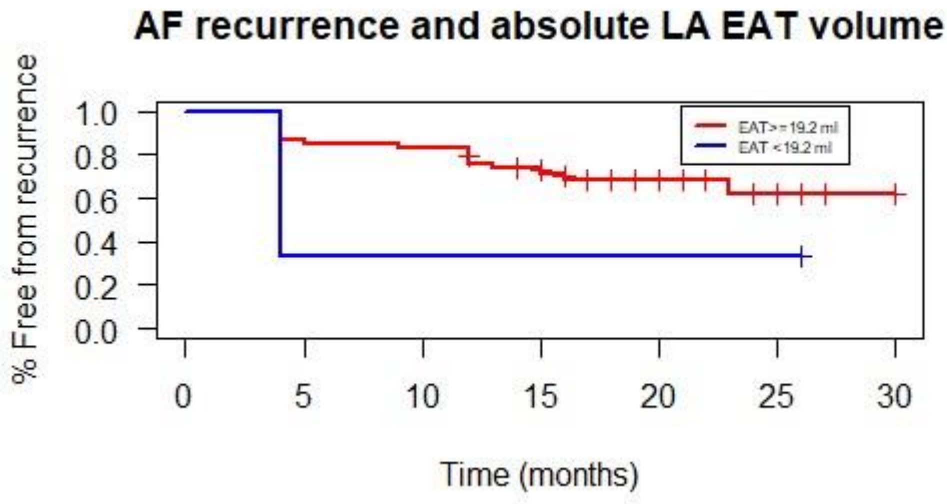

Supplement: Supplementary file 1 [file jcdd-11-00137-s001.zip › jcdd-2950303-supplementary.pdf]
